# Functional Plant-Based Beverage Fortified with Hazelnut Cuticle Polyphenols: Antioxidant and Phenolic Content Characterization

**DOI:** 10.3390/molecules30030433

**Published:** 2025-01-21

**Authors:** Raffaele Conte, Fabrizia Sepe, Sabrina Margarucci, Ezia Costanzo, Orsolina Petillo, Gianfranco Peluso, Loredana Marcolongo, Anna Calarco

**Affiliations:** 1 Research Institute on Terrestrial Ecosystems (IRET), CNR, Via Pietro Castellino 111, 80131 Naples, Italy; raffaele-conte@cnr.it (R.C.); fabrizia.sepe@iret.cnr.it (F.S.); sabrina.margarucci@cnr.it (S.M.); ezia.costanzo@iret.cnr.it (E.C.); orsolina.petillo@cnr.it (O.P.); gianfranco.peluso@unicamillus.org (G.P.); anna.calarco@cnr.it (A.C.); 2 National Biodiversity Future Center (NBFC), 90133 Palermo, Italy; 3 Department of Veterinary Medicine and Animal Production, University of Naples, Federico II, Via Federico Delpino 1, 80137 Naples, Italy; 4 Faculty of Medicine and Surgery, Saint Camillus International University of Health Sciences, Via di Sant’Alessandro 8, 00131 Rome, Italy

**Keywords:** natural deep eutectic solvents (NADESs), plant by-products, hazelnut cuticles, bioaccessibility

## Abstract

In recent decades, there has been growing interest in the fortification of food products with antioxidants and phenolics derived from plant by-products. The present study focused on the production of a plant-based beverage enriched with hazelnut cuticle extract to characterize its antioxidant content, phenolic profile, and organoleptic characteristics. Liquid chromatography-mass spectrometry (LC-MS) enabled the identification of key polyphenols in hazelnut cuticles, including catechin, epicatechin, and quercetin derivatives, guiding the selection of a biocompatible Natural Deep Eutectic Solvent (NADES) composed of choline chloride and lactic acid for efficient extraction. The obtained phytochemical profile of the extract revealed a high concentration of bioactive compounds, with a Total Phenolic Content of 160.88 ± 14.27 mg GAE/g and Antioxidant Power measured by DPPH of 5848.2 ± 11.3 μmol TE/g. The bioaccessibility of phenolics in the fortified hazelnut-based beverage was determined after in vitro digestion, reaching a value of 89.7%, indicating excellent release and stability during digestion. Organoleptic evaluation revealed high sensory acceptability, with aftertaste scoring 3.61 ± 0.4 respect the 3.94 ± 1.3 result of reference milk, on a 5-point scale. In conclusion, this study demonstrates the potential for sustainable valorization of hazelnut cuticles, through their incorporation as NADES extracts in plant-based milk, providing an innovative solution to reduce food waste while catering to consumer demand for nutritionally enriched and eco-friendly products.

## 1. Introduction

Over the past decade, consumer preferences have significantly influenced global food supply chains. A marked shift has occurred from synthetic to natural food ingredients, alongside a growing demand for products with high nutritional value. This shift has driven the food industry to increasingly incorporate plant-based resources, such as fruits, vegetables, herbs, and spices, into food products [1,2]. However, the rising demand for these resources has also generated substantial food waste, including peels and seeds, raising concerns about their environmental impact and management [3,4]. 

Several research papers have highlighted that these plant-derived by-products are rich in antioxidants, phenolic compounds, dietary fibers, and other bioactive compounds [5,6]. Consequently, their potential use as ingredients for food fortification and the development of functional foods has been extensively explored [7,8]. Many natural antioxidants have been incorporated into food products to enhance flavor, aroma, and color, as well as for their antimicrobial and therapeutic properties [9,10]. However, consumer acceptance is highly dependent on the sensory and organoleptic characteristics of foods, making it essential to evaluate these properties in final functional food products [11]. Then, in response to the growing consumer interest in healthier food options, the food industry has increasingly focused on developing a variety of functional foods that meet both nutritional and sensory expectations [12]. Hazelnut cuticles, a by-product of the nut industry, are an exceptional source of polyphenols, including gallic acid, caffeic acid, p-coumaric acid, ferulic acid, quercetin-3-*O*-rutinoside, catechin, epigallocatechin, kaempferol-3-*O*-glucoside, quercetin, and apigenin [13]. Remarkably, the polyphenol content in hazelnut cuticles can exceed that of the kernel by 10–20 times, making them highly suitable for valorization in nutraceuticals and functional foods [14]. Indeed, these bioactive compounds are renowned for their antioxidant and anti-inflammatory properties, yet their effective extraction, stability, and incorporation into food matrices remain challenging due to their polarity and quantification difficulties. 

Natural Deep Eutectic Solvents (NADESs) have emerged as a promising green technology for the extraction of antioxidants, particularly polyphenols, due to their ability to mimic natural intracellular environments. Their exceptional solvating power enables the extraction of a wide range of bioactive compounds, including those that are poorly soluble in conventional solvents like water or ethanol. Moreover, NADESs are non-toxic, biodegradable, and based of food-grade components, making them ideal for food and nutraceutical applications. Unlike traditional solvents, NADESs eliminate the need for post-extraction purification, as their biocompatibility allows them to be directly incorporated into final products, reducing processing time and costs [15]. Therefore, this study evaluates the feasibility of using the polyphenol-enriched extract as an aqueous phase in the production of vegetable milk derived from hazelnut. In addition, the composition and antioxidant activity of polyphenol-rich NADES extracts and the ability of such extracts to improve the stability of the final product while preserving its acceptability to the final consumer were evaluated. This approach would allow the development of a functional food product with enhanced health properties and improved antioxidant activity, while meeting several commercial needs such as prolonged stability of polyphenols and reduction of the high cost of the industrial extraction process.

## 2. Results and Discussion

### 2.1. Characterization of Natural Deep Eutectic Solvent (NADES) and Selection of Suitable Solvent for Polyphenols Extraction

Natural Deep Eutectic Solvents (NADESs) offer excellent environmentally friendly alternatives to organic solvents for the extraction of natural products due to their biodegradability and biocompatibility [15]. NADESs are typically composed of a hydrogen bond donor (HBD) mixed with a hydrogen bond acceptor (HBA) leading to eutectic mixture characterized by their versatility and ability to dissolve a wide range of compounds [16]. Moreover, NADES formulations are made from components which are naturally occurring in the environment, and no studies indicate dangerous or negative consequences [17].

Several research report the efficacy of choline chloride-based NADES in the extraction of phenolic chemicals [18]. Choline chloride (ChCl, 90%) is a cost-effective, biodegradable, and non-toxic quaternary organic salt that served as a HBA with various compounds, including sugars (glucose, sucrose), and acids (lactic acid, malic acid, and citric acid) as HBD. Among the acid-based solvents, NADESs composed of malic acid and lactic acid demonstrated the highest polarity [19] a feature particularly well-suited for selectively extracting highly polar polyphenols with stronger antioxidant activity [20]. Viscosity also played a critical role in the extraction efficiency. Lactic acid-based NADESs were characterized by good fluidity and low viscosity, as previously reported by Altamash et al. [21]. In contrast, malic acid-based NADES exhibited higher viscosity and poorer fluidity [21]. Additionally, Xin et al. [22] noted that increasing the proportion of choline chloride in NADES formulations results in higher viscosity, which can hinder liquid flow. On the other hand, hydroalcoholic solvents, known for their high efficiency in extracting polyphenols from plant by-products [23,24], owe part of their success to optimal viscosity, which enhances permeability through plant cell walls [25]. Preliminary results obtained in our laboratory indicated that acid-based NADESs exhibit superior extraction efficiency for phenolic compounds compared to sugar-based NADES (Table S1). The superior extraction efficiency of acid-based NADESs compared to sugar-based NADESs for phenolic compounds can be attributed to several factors. Acid-based NADESs have stronger hydrogen-bonding capabilities and higher polarity, which enhance their ability to dissolve phenolic compounds, typically polar in nature. Additionally, the acidic environment of these solvents can ionize phenolic hydroxyl groups, increasing their solubility and promoting better interactions with the solvent. Acid-based NADESs also tend to have lower viscosity than sugar-based NADESs, facilitating improved mass transfer during extraction. Furthermore, the chemical structure and functional groups of acid-based NADESs often show better compatibility and affinity for phenolic compounds, aiding in solubilization. The acidic conditions may also stabilize phenolic compounds through protonation, enhancing their recovery. Based on these observations, in the present study, critical parameters such as the molar ratio between choline chloride and acidic hydrogen bond donors, as well as synthesis temperature, were optimized to achieve the best balance of viscosity and polarity for efficient polyphenol extraction (Table 1).

NADES with a 1:1 ChCl:HBD ratio were overly viscous and less homogeneous, resulting in low total phenolic content. In contrast, a 1:2 ChCl:HBD ratio provided better homogeneity and stability, while a 1:3 ChCl:HBD ratio exhibited lower viscosity but, at times, insufficient homogeneity. Regardless of the synthesis temperature, total phenolic content (TPC) data indicate that temperature is not a determining factor. Notably, Code 2 (ChCl:LA 1:2 ratio, 60 °C) achieved the highest TPC (160.88 ± 14.27 mg GAE/g) with manageable viscosity (1.56 mPa·s at 25 °C), while Code 12 (ChCl:MA 1:3 ratio, 80 °C) also performed well with a slightly lower TPC (147.56 ± 13.11 mg GAE/g) but excellent stability and low viscosity (2.65 mPa·s at 25 °C). In addition, both presented values of degradation rate of 0.17, 0.23 and 0.27 at −18 °C, 4 °C and 25 °C, respectively. Based on these results, Codes 2 and 12 (from this moment onwards defined as NADES1 and NADES2, respectively) were selected for subsequent experiments.

### 2.2. Extraction of Polyphenols from Roasted Hazelnut Cuticles

Hazelnut cuticles, a by-product of nut processing, are an underutilized yet exceptionally rich source of polyphenols, making them a promising candidate for valorization in nutraceutical and functional food industries [26]. These cuticles are particularly abundant in flavonoids, including catechins, epicatechins, and oligomeric procyanidins, as well as phenolic acids such as gallic, caffeic, ferulic, and p-coumaric acids [13]. Compared to the hazelnut kernel, the polyphenol content in the cuticles is significantly higher, with some studies reporting concentrations 10–20 times greater [14]. As reported by Ghirardello et al. [27], the total phenolic content (TPC) of hazelnut cuticles can reach several hundred milligrams of gallic acid equivalents (GAE) per 100 g, depending on factors such as cultivar, geographical origin, and processing conditions [27]. In particular, Although processing methods, particularly roasting, significantly impact the polyphenol profile with a reduction of up to 50% of the phenolic content, on the other hand enhance the bioavailability of certain compounds due to the transformation of complex structures into more bioactive forms [28]. In addition, the polyphenol content is highly influenced by the solvent [29].

In recent years, Natural Deep Eutectic Solvents (NADESs) have emerged as innovative, greener and safer alternatives to conventional organic solvents. NADESs are particularly effective for extracting polyphenols from vegetables due to their ability to form strong hydrogen bonds with phenolic compounds. This interaction not only enhances extraction efficiency but also protects biomolecules from degradation [30,31,32,33,34,35,36]. Furthermore, NADESs have been shown to extract a higher concentration of polyphenols and tannins, thereby improving the antioxidant and scavenging capacities of hazelnut cuticle extracts [13,30]. These advantages, combined with the already high polyphenol content in hazelnut cuticles, make NADESs a promising tool for sustainable and efficient phenolic compound extraction.

In this study, liquid chromatography-mass spectrometry (LC-MS) was employed, to thoroughly investigate and compare the extraction efficiency of a series of NADES and conventional solvents (such as water and ethanol) for the extraction of hazelnuts polyphenols. To the best of our knowledge, this is the first time that such comparative analysis has been conducted specifically on roasted hazelnut cuticles. By leveraging LC-MS, the aim is to achieve a comprehensive profiling of the extracted compounds, providing detailed insights into their concentration and possible variations based on the solvent used.

The phenolic profiles of all samples were elucidated by LC-MS analysis. As shown in Table 2, the phenolic profile was similar between water, water/ethanol, NADES1 and NADES2 extracts, revealing the presence of four phenolic acids (gallic acid, caffeic acid, p-coumaric acid, and ferulic acid) and six flavonols, including quercetin, catechin, epigallocatechin, quercetin-3-*O*-rutinoside, kaempferol-3-*O*-glucoside, and apigenin. Among the phenolic acids detected, gallic acid and caffeic acid had the highest concentrations, followed by p-coumaric acid and ferulic acid. However, gallic acid emerged as the predominant phenolic acid overall, accounting for 95.0% of the total polyphenolic compounds and more than 98.0% of the phenolic acids on average. It is noteworthy that the coefficient of variation for the relative amount of major phenolic compounds between NADES1 and Ethanol 80% in Water was lower than 10% of respective mean, indicating no relevant differences between the two extracts.

**Figure 1:**
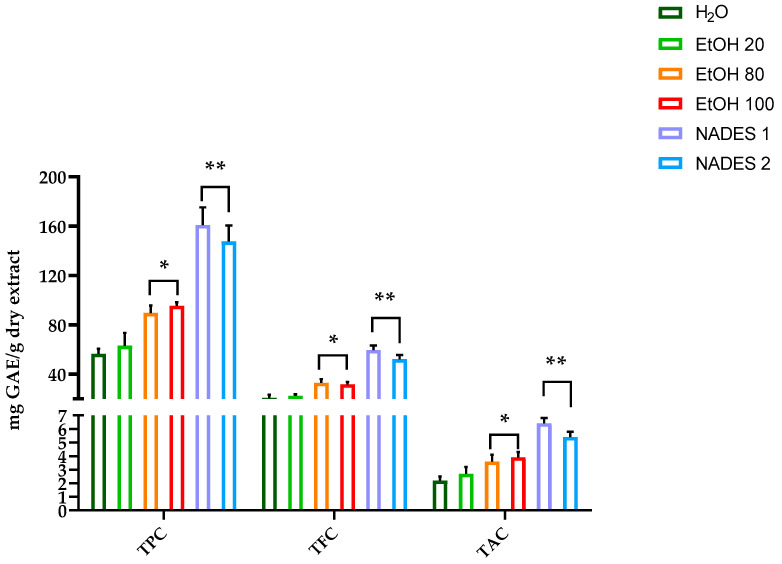
Effect of type of solvent on Total Phenolic Content (TPC), Total Flavonoid Content (TFC), and Total Anthocyanin Content (TAC). For each sample, six different experiments were carried out, and the results are expressed as the means of the values obtained (mean ± SD). Statistically significant variations: * *p* ≤ 0.01 and ** *p* ≤ 0.005 versus H_2_O and EtOH 20. GAE refers to gallic acid equivalents.

Figure 1 also demonstrates that the highest TPC and TFC contents were recorded by NADES1 followed by NADES2 compared with ethanol aqueous solutions, while no significant difference was detected for TAC. This can be attributed to the unique solvent properties of NADES, which are known to enhance the solubility and stability of polyphenolic compounds due to the strong hydrogen bonding interactions between the components of the solvent and the polyphenols [37].

### 2.3. Antioxidant Capacity of Hazelnut Cuticles

Antioxidant compounds may be incorporated into human and animal diets to mitigate the excessive generation of reactive oxygen species (ROS), which contribute to oxidative stress, a condition associated with various diseases, including cancer, diabetes, atherosclerosis, arthritis, neurodegenerative disorders, and premature aging [28]. Furthermore, they serve as food preservatives, mitigating oxidative damage in foodstuffs and so enhancing their quality and shelf life [29].

The evaluation of antioxidant activity in natural extracts can be performed using complementary approaches and methodologies. The chemical-based assays can be divided into two distinct mechanisms: those based on the scavenging activity toward a stable free radical (DPPH, ABTS) and those able to reduce or chelate metal ions (FRAP, CUPRAC). In this study, the antioxidant capabilities of NADES extracts were assessed using both approaches due to their capacity to target many unique processes, facilitating a thorough valuation of the antioxidant potential of hazelnut cuticle extracts [38,39]. Results were compared with those of the extracts obtained by using water, ethanol (EtOH100), and ethanol:water mixture (80:20 EtOH80, and 20:80 EtOH 20).

**Figure 2:**
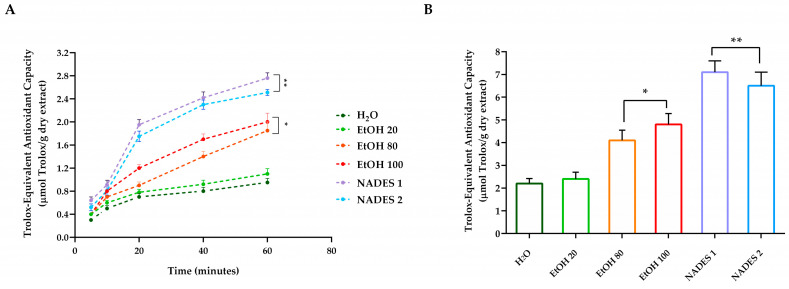
Hazelnut cuticles antioxidant-reducing capacity. (**A**) FRAP assay and (**B**) CUPRAC assay. For each sample, six different experiments were carried out, and the results are expressed as the means of the values obtained (mean ± SD). Statistically significant variations: * *p* ≤ 0.01 and ** *p* ≤ 0.005 versus H_2_O and EtOH 20.

As shown in Figure 2, NADES1 and NADES2 were more efficient in terms of antioxidant activity with respect to water and EtOH 20. In particular, the ability of hazelnut cuticles to reduce iron ions was significantly higher (*p* < 0.005) in both NADES extracts (Figure 2A,B) when compared to canonical extractions (H_2_O, EtOH 20).

**Figure 3:**
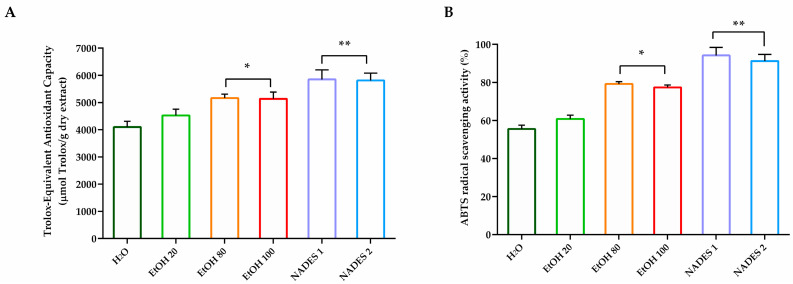
In vitro antioxidant activity of hazelnut cuticles extracts. (**A**) DPPH assay. Total antioxidant activity is expressed as micromoles of Trolox equivalents (TE) per gram of dried extract. EC50 was read as micromoles of Trolox equivalents (TE) of dried extract required to scavenge the initial DPPH radical by 50%. Data are expressed as the means (the standard deviation (*n* = 3) of extracts. (**B**) ABTS radical scavenging activity. For each sample, six different experiments were carried out, and the results are expressed as the means of the values obtained (mean ± SD). Statistically significant variations: * *p* ≤ 0.01 and ** *p* ≤ 0.005 versus H_2_O and EtOH 20.

The antioxidant activity of the hazelnut cuticle samples, measured using the DPPH method, was highest for the NADES 1 extract, followed by NADES 2 (5848.2 and 5810.5 µmol TE eq/g of dried extract, respectively), showing a better scavenging activity compared with the extracts obtained using the conventional methodologies (Figure 3A). The ABTS assay showed the highest amount of antioxidants with the extraction using NADES1 (Figure 3B). Furthermore, the solution achieved with NADES1 allowed the highest scavenging activity to be obtained even at lower extract concentrations than those reached with the extractions carried out with conventional solvents. A general trend of a dose-dependent effect was found for each solvent and mixture used to extract bioactive molecules. Overall, the antioxidant-activity trend observed in hazelnut cuticles extractions, as determined by DPPH, ABTS, FRAP and CUPRAC assays, showed a positive correlation with the amount of polyphenols, suggesting that these compounds are responsible for most of the antioxidant effect [40].

**Figure 4:**
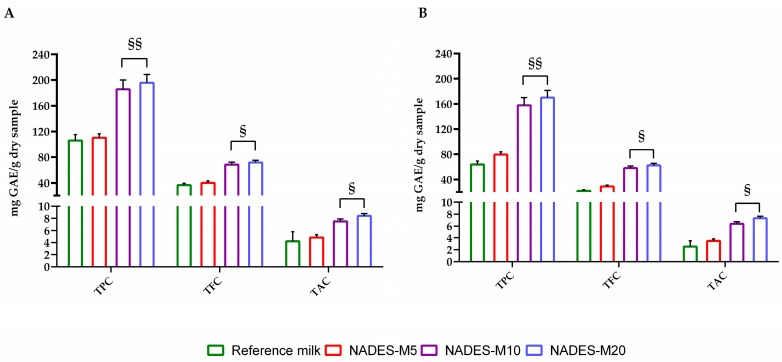
Total Phenolic Content (TPC), Total Flavonoid Content (TFC), and Total Anthocyanin Content (TAC) of reference milk or NADES fortified milk. (**A**) represents the start of the storage period, whereas (**B**) illustrates the conditions after 30 days. For each sample, six different experiments were carried out, and the results are expressed as the means of the values obtained (mean ± SD). Statistically significant variations: § *p* ≤ 0.01 and §§ *p* ≤ 0.005 versus Reference milk and NADES-M5. GAE refers to gallic acid equivalents.

Based on these results, fortified beverages were prepared by incorporating NADES1 extracts into freshly produced nut milk at concentrations of 5%, 10%, and 20% (*w*/*w*), labeled as NADES-M5, NADES-M10, and NADES-M20, respectively. As expected, incorporation of NADES extracts into beverage improved the phenolic (TPC), flavonoid (TFC) and anthocyanin (TAC) content compared to reference milk (Figure 4A). Among the fortified beverage, NADES-M10 and NADES-M20 showed the highest polyphenols content (185.6 and 195.6 mg GAE/g dry sample, respectively), whereas the addition of 5% of extract does not substantially change the polyphenol content compared with the control (110.4 mg GAE/g dry sample vs. 106.3 mg GAE/g dry sample). The same tendency is observed in the case of TFC and TAC. At the end of storage period (30 day, 25 °C), a decrease of 15–30% of TPC, TFC and TAC were recorded (Figure 4B), with the lowest reduction for NADES-M20 (13%) and the highest for Reference milk.

**Figure 5:**
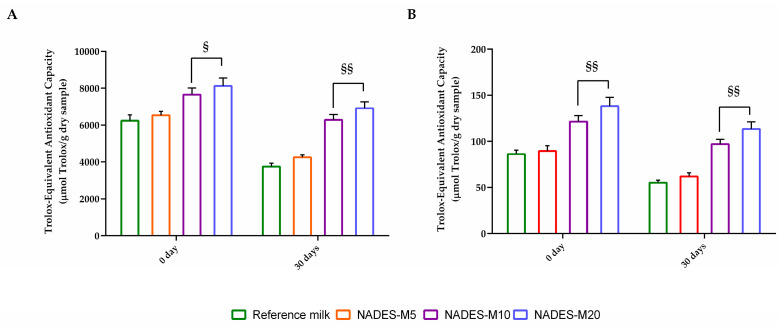
In vitro antioxidant activity of fortified milk. (**A**) DPPH assay. Total antioxidant activity is expressed as micromoles of Trolox equivalents (TE) per gram of dried sample. Data are expressed as the means (the standard deviation (n = 3) of samples). (**B**) ABTS radical scavenging activity. For each sample, six different experiments were carried out, and the results are expressed as the means of the values obtained (mean ± SD). Statistically significant variations: § *p* ≤ 0.01 and §§ *p* ≤ 0.005 versus Reference milk and NADES-M5.

As shown in Figure 5, the antioxidative activity of the samples measured using DPPH and ABTS assay decreased during the whole period of storage. The results indicated that reference milk had about 40% DPPH and ABTS radical scavenging activity loss after one month of storage, whereas the antioxidative activity loss reported for NADES-M10 and NADES-M20 is ~18% higher respect to the initial storage period (Table 3).

### 2.4. Functional Vegetable Milk Properties and Bioaccessibility

In contemporary society, plant-based beverages are perceived not merely as thirst quenchers but as newer alternatives to healthy foods. The functionality of these beverages may meet many requirements and lifestyles—enhancing energy, combating aging, alleviating weariness and stress, and targeting certain ailments, while the sector continues to expand. A significant practical requirement is the provision of milk alternatives to address issues related to cow milk allergy, lactose intolerance, or caloric concerns [41]. In addition, plant-based beverages are fortified with key nutrients such as calcium, iodine, vitamin B12 and vitamin D to improve human health. However, to be effective, phenolic compounds must be liberated from the plant matrix during digestion in a bioaccessible form for absorption by the body. Nonetheless, the assimilation of numerous bioactive substances is impeded by various physicochemical and physiological processes occurring in the gastrointestinal tract (GIT) subsequent to their ingestion. The application of NADESs can improve the solubility and stability of phenolic compounds, hence increasing their availability for absorption. Choline chloride, lactic acid, and malic acid are components of the NADES formulation, and all have been approved as food additives. The addition of these compounds does not introduce any additional allergenic risk, as these molecules are food-grade and generally recognized as safe by the FDA when used in regulated amounts [42,43]. In the present study, the concentrations of these components in the plant-based milk fortified with hazelnut cuticles NADES extract (NADES milk) were carefully maintained under the value of 1% *w*/*v* to ensure compliance with the FDA “Code of Federal Regulations Title 21” prescribed limits for food additives. For comparison, a commercial-like formulation was replicated using the same recipe. No solvent was eliminated prior to the utilization of the extract, and no toxicity was detected for the NADES itself, indicating that this NADES formulation can be used to produce ready-to-use extracts, as they consist of food-grade components that seemingly do not require removal prior to use [42,43,44].

The sensory acceptability of NADES fortified milk beverage was evaluated on the basis of physical appearance and overall acceptability. Both milk exhibited a smooth, creamy texture, and were free of any undesirable separation, indicating that the emulsification process was successful. The aftertaste and milkiness of NADES milk were slightly lower at a rating value of 3.61 ± 0.4 and 2.94 ± 0.6, respectively, as compared with Reference milk at a rating of 3.94 ± 1.3 and 3.26 ± 1.1, respectively. Panelists’ perception toward creaminess of NADES milk and Reference milk were almost similar with a score of 3.25 ± 0.6 and 3.22 ± 0.9, respectively. Overall, there were no significant differences in all attributes of milkiness, creaminess, mouthfeel, color and aftertaste between both prepared milk, suggesting that use of the NADES extract did not negatively impact the sensory qualities of the final product.

To evaluate the bioaccessibility of polyphenols in NADES milk and the influence of the extraction matrix, the samples were subjected to an in vitro gastrointestinal digestion protocol [45,46,47]. Following digestion, the total phenolic content (measured as gallic acid concentration) of the plain NADES1 extract decreased significantly (*p* < 0.05) from 6467.45 ± 363.09 to 4903.84 ± 122.93 mg of GAE/g. The bioaccessibility index, calculated as the ratio of the total phenolic content after digestion to the initial content, was determined to be 75.8%.

As reported by da Silva et al. [44], the bioavailability of phenolic substances can be improved by choosing suitable NADES formulations. The combination of choline chloride, glycerol, and citric acid as NADESs serves as an effective vehicle for enhancing the oral absorption of bioactive compounds, such as anthocyanins, resulting in a 140% increase in oral bioavailability of anthocyanins in rats compared to extracts obtained via organic solvents. Proline–glutamic acid (2:1) demonstrated efficacy in enhancing rutin bioavailability; proline–malic acid–lactic acid combinations markedly elevated plasma berberine levels post-oral administration of three NADESs in mice, in contrast to the results obtained with water suspension after 30 min [48].

Regarding the fortified food matrices (Table 4), in NADES milk, the total phenolic content after digestion reached 1790.68 ± 15.63 mg GAE/g of milk, while the reference milk resulted in a value of 72.15 ± 2.88 mg GAE/g. The corresponding bioaccessibility indexes is 89.7% for NADES milk and 73.8% for the Reference milk. The observed differences in the amount (mg) of polyphenols can be attributed to the superior efficiency of the NADES extraction method. Even when equal amounts of hazelnut cuticles are used to prepare the milk, the NADES extraction likely facilitates a more effective release and solubilization of polyphenols, maximizing their incorporation into the final product. This efficiency underscores the potential of NADES in improving the functional and nutritional properties of food formulations. Analyzing singularly each stage of the digestion protocol, all phases facilitate the release of polyphenols from the produced milk. In the oral stage, minimal loss of polyphenols was observed, indicating that the initial breakdown of the food matrix during mastication did not significantly affect the polyphenolic content. During the gastric phase, where pepsin and acidic conditions prevail, a slight decrease in polyphenol concentration was noted. However, the majority of polyphenols remained in the soluble fraction, suggesting that the gastric conditions did not result in substantial degradation or loss of bioactive compounds. However, the most significant absorption of polyphenols must occur during the intestinal phase, where the digestive enzymes, bile salts, and a neutral pH favored the further solubilization and absorption of polyphenols. Also in this stage, a substantial proportion of polyphenols is being available, as shown in Table 4. These findings underscore the efficiency of the gastrointestinal digestion process in liberating polyphenols from NADES milk, highlighting the potential of hazelnut cuticle extracts as a source of bioactive compounds with high bioavailability. However, the bioavailability of benzoic and hydroxycinnamic acids, commonly found in hazelnut cuticles, is generally considered low due to their limited intestinal absorption [49]. Instead, a significant portion reaches the colon, where they undergo metabolism by the gut microbiota [46]. Despite this, hydroxycinnamic acids can exert localized biological activity within the gastrointestinal tract prior to absorption, functioning as anti-inflammatory and anti-proliferative agents and offering protective effects against conditions such as inflammatory bowel disease and colon cancer [46]. In such cases, their bioaccessibility—rather than bioavailability—is critical, requiring stability and effective release during gastrointestinal digestion. Overall, when compared to a commercial-like reference formulation, the NADES-fortified hazelnut milk exhibits several advantages. The use of NADES extract enhances the bioavailability of phenolic compounds, addressing the challenge of poor absorption of bioactive substances in the gastrointestinal tract. Sensory evaluation also revealed that the NADES milk performed comparably to the reference milk in terms of creaminess, mouthfeel, and overall acceptability. However, minor differences were observed in aftertaste and milkiness, where the NADES milk scored slightly lower. These differences, although not significant, highlight areas for potential improvement in product optimization. In conclusion, the use of extracts obtained by NADESs to fortify foods such as vegetable milk allows a reduction in processing complexity, eliminating the need to remove the solvent, while preserving the nutritional and functional properties of the extract.

## 3. Materials and Methods

### 3.1. Materials

Choline chloride (ChCl, ≥99% purity), lactic acid (LA, ≥85% purity), malic acid (MA, ≥85% purity), gallic acid, rutin hydrate, quercetin, cyanidin-3-*O*-glucoside, 2,2-diphenyl-1-picrylhydrazyl (DPPH•), 2,2′-azino-bis(3-ethylbenzothiazoline-6-sulfonic acid) (ABTS), Trolox, ammonium acetate, iron(III) chloride (FeCl_3_), potassium persulfate (K_2_S_2_O_8_), ethanol (<99.5% purity), sodium acetate, aluminum chloride, sodium carbonate (Na_2_CO_3_), simulated digestive enzymes (amylase, pepsin, pancreatin), bile salts, and all other reagents were obtained from Sigma-Aldrich (Milan, Italy). Polyphenols standards Gallic Acid 149-91-7 catalog number G7384, Caffeic Acid 331-39-5 catalog number 205546, p-Coumaric Acid 501-98-4 catalog number C9008, Ferulic Acid 1135-24-6 catalog number F9008, Quercetin-3-*O*-Rutinoside (Rutin) 153-18-4 catalog number 16654, Catechin 154-23-6 catalog number 0365, Epigallocatechin 989-51-5 catalog number 0366, Kaempferol-3-O-Glucoside 528-59-0 catalog number 68437, Quercetin 117-39-5 catalog number 00200595, Apigenin 520-36-5 catalog number 0367 were purchased from Sigma Aldrich. All chemicals and solvents were of analytical grade and used as received without further purification. Roasted Skins from hazelnuts (*Corylus avellana* L.; grown in the Mountain Community of the Vallo Di Lauro and Baianese Area of Campania region) were kindly supplied by Francesco Basile Co SRL, freeze-dried (Alpha 1-2 LD plus Christ/Sigma, Osterode am Harz, Germany) for two days, milled to a particle size suitable for commercial applications (less than 100 μm), and stored at 25 °C in a desiccator until extract preparation.

### 3.2. Preparation and Characterization of NADES

Choline chloride was dried in a vacuum concentrator (Savant SPD131DDA SpeedVac129 Concentrator, Thermo Scientific, Milan, Italy) at 60 °C for 24 h before use. NADESs were synthetized at certain molar ratios of ChCl and hydrogen bond donor acids (LA or MA). The mixtures were heated at temperatures ranging from 60 °C to 80 °C in presence of MilliQ water in a water–NADES (*v*/*v*) percentage of 33%. The solution was stirred until a homogeneous and transparent liquid was obtained. Heating was maintained for approximately 30 min to ensure complete dissolution and interaction between the components. The resulting NADESs were allowed to cool to room temperature and were stored in an airtight container to prevent moisture absorption. Formation of the eutectic mixtures was confirmed by the absence of crystalline residues and the presence of a stable, viscous liquid at room temperature.

### 3.3. Viscosity Measurement

The viscosity of the prepared choline chloride–lactic acid (ChCl–LA) or choline chloride–malic acid (ChCl-MA) NADES was measured using a rotational viscometer (Brookfield Dial Viscometer; AMETEK, Berwyn, PA, USA) equipped with a spindle for low- to medium-viscosity fluids. Prior to measurement, the viscometer was calibrated using standard viscosity fluids to ensure accuracy. Approximately 20 mL of each NADES sample was transferred to a clean, temperature-controlled sample holder. The spindle was carefully immersed in the sample without introducing air bubbles. Viscosity measurements were conducted at temperatures of 25 °C and 50 °C to mime the storage and utilization conditions. The spindle rotation speed was adjusted to ensure stable torque readings, and measurements were recorded after the system reached equilibrium. Each viscosity value was determined in triplicate to ensure reproducibility, and the mean value was reported in milliPascal-seconds (mPa·s). Between measurements, the spindle and sample holder were thoroughly cleaned with deionized water and dried to prevent contamination.

### 3.4. Extraction of Polyphenols from Roasted Hazelnut Cuticles

Solid–liquid ratios of 0.1 g of freeze-dried roasted hazelnut cuticles per mL of prepared NADES, or different water–ethanol ratio (*v*/*v*), were used for extraction. The samples were incubated at 50 °C for 2 h under constant stirring, then centrifuged for 10 min at 2570 rcf (OHAUS FC5718R centrifuge with 19/005 rotor). The supernatant was decanted, filtered through Whatman No. 1 filter paper and stored at 4 °C until further LC-MS analyses were performed (Section 3.9).

### 3.5. Stability of Prepared Extracts

The storage stability of the prepared extracts was measured according to Dai et al. [50]. Polyphenol concentration was specifically used as a parameter to evaluate the stability of the extract, as it reflects the retention of bioactive compounds during storage [50]. Briefly, extracts were stored in the dark at 25 °C, 4 °C and −18 °C for 60 days, and were monitored and analyzed over the 30 days using LC-MS (Section 3.9). Data are expressed as degradation rate (1 − C/C_0_), where C_0_ is the initial polyphenols concentration and C is the polyphenols concentration after storage. In addition, stability of NADES milk was assessed through the antioxidative activity measured using DPPH and ABTS assay during the whole period of storage. This parameter provides a functional measure of the bioefficacy of the fortified milk over time.

### 3.6. Total Phenolic Content (TPC)

The total phenolic content (also called Folin assay) of the hazelnut cuticle extracts and different NADES milk samples was determined using the Folin–Ciocalteu colorimetric method [51]. Briefly, 100 µL of each extract was mixed with 500 µL of Folin–Ciocalteu reagent (diluted 10-fold with distilled water). After 5 min of incubation at room temperature, 1.5 mL of a 20% sodium carbonate (Na_2_CO_3_) solution was added to the mixture. The solution was then incubated for 1 h in the dark at room temperature to allow the development of a blue color. The absorbance of the resulting solution was measured at 765 nm using a microplate reader Cytation 5 Cell Imaging Multimode Reader (BioTek, Bad Friedrichshall, Germany). A calibration curve was prepared using gallic acid (GA) as the standard within the concentration range 0.01–0.8 mg/mL (equivalent to a range 0.5–350 mg of polyphenol), and the results were expressed as mg of gallic acid equivalents (GAE) per gram of dry extract (referred as the cuticle content of each extract). All measurements were performed in triplicate, and the results were averaged for each extract.

### 3.7. Total Flavonoid Content (TFC) Assay

The total flavonoid content (TFC) of the hazelnut cuticle extracts and different NADES milk samples was determined using a colorimetric aluminum chloride method as described by Nurcholis et al. [52], with slight modifications. Fifty microliters of the sample, positive control (rutin hydrate), calibration standard (0.5 mg/mL quercetin stock solution), and blank (ethanol) were dispensed into a 96-well microtiter plate in technical triplicate. Each well was supplemented with 130 µL of ethanol, followed by 20 µL of a 1:1 (*v*/*v*) mixture of 10% aqueous aluminum chloride solution and 1 mol/L sodium acetate solution. The plate was shaken for 12 s and incubated at room temperature for 40 min. After incubation, the absorbance was recorded at 415 nm using a microplate reader. The standard solutions of quercetin were prepared by serial dilutions (5–100 μg/mL). The concentration of total flavonoid content in the test samples was calculated from the calibration curve (Y = 0.0012x + 0.0031, R^2^ = 0.998) and expressed as mg gallic acid equivalents (GAE)/g of dried extract. All determinations were performed in triplicate.

### 3.8. Total Anthocyanin Content (TAC) Assay

The total monomeric anthocyanin content (TAC) was measured using the pH differential method as described by Giusti and Wrolstad [53]. Sample dilutions were prepared using pH 1.0 and pH 4.5 buffers, and their absorbance values were measured at 520 nm and 700 nm with a microplate reader. The monomeric anthocyanin content was calculated using the formula:

$$TAC\;\left(\frac{mg}{L}\right)\;=\;\frac{A\;\times \;MW\;\times \;DF\;\times \;1000}{\varepsilon \times l}$$

where A represents the absorbance difference between (A_520_–A_700_) at pH 1.0 and (A_520_–A_700_) at pH 4.5, MW is the molecular weight of cyanidin-3-*O*-glucoside (449.2 g/mol), DF is the dilution factor, 1000 is the conversion factor from g to mg, ε is the molar extinction coefficient (26,900 L/mol·cm), and l is the path length (1 cm).

### 3.9. Identification and Quantification of Phenolic Compounds by LC-MS

Polyphenol quantification was performed using a Shimadzu LCMS-8060 triple quadrupole mass spectrometer coupled with a high-performance liquid chromatography (HPLC) system. Separation was achieved using a Kinetex C18 column (100 × 4.6 mm, 2.6 µm; Phenomenex, Torrance, CA, USA) maintained at 40 °C. The mobile phase consisted of acetonitrile and water with 0.1% formic acid in an isocratic ratio of 30:70 (*v*/*v*). The flow rate was set at 0.3 mL/min, and the injection volume was 25 µL. Mass spectrometric detection was performed in both positive and negative electrospray ionization (ESI) modes, with the following optimized parameters: ion source temperature, 300 °C; desolvation line temperature, 250 °C; and nebulizing gas flow, 3.0 L/min. Data acquisition was conducted in single ion monitoring (SIM) mode, targeting specific polyphenolic compounds based on their mass to charge ratio (Table S2). Calibration curves for identified polyphenols were prepared using authentic standards to enable quantification. Data were processed using LabSolutions software (Version 5.118), and peaks were identified by comparing retention times and mass spectra with those of standards or of an internal developed database.

### 3.10. Determination of Antioxidant Activity

#### 3.10.1. ABTS Radical Scavenging Activity

The antioxidant capacity was evaluated as in vitro scavenging activity against the radical-cation 2,2–Azino-bis(3-ethylbenzothiazoline-6-sulfonic acid) (Sigma–Aldrich) according to Re et al. [54] with slight modifications. The ABTS radical cation (ABTS•+) was produced by reacting 2,2′-azino-bis(3-ethylbenzothiazoline-6-sulfonic acid) (ABTS) with potassium persulfate solution as described in the reference material and kept in a dark place for 16 h at room temperature. The ABTS•+ solution was diluted in demineralized water until absorbance was 0.7 ± 0.05 at 734 nm. A 30 μL of diluted extract (1:10–1:500) or Trolox diluted in ethanol was mixed with 270 μL of the ABTS•+ radical solution. The reaction was performed for 20 min in the dark at room temperature. The absorbance was measured at 734 nm with a Cytation 3 Reader. Each sample was run in triplicate. Results were expressed as percentage of scavenging activity for dry extract (Figure 3) and as μmol Trolox equivalent (TE)/g dry sample (Figure 5), where “dry extract” refers to the freeze-dried roasted hazelnut cuticles extracts while “dry sample” refers to the dried vegetable milk.

#### 3.10.2. DPPH Radical Scavenging Activity

Antioxidant activity of extracts was determined following the protocol published by Di Cristo et al. [55] employing DPPH (2,2-diphenyl-1-picrylhydrazyl; Sigma Aldrich). Trolox was used as a standard. The results were expressed µmol of Trolox equivalent per g of dry extract or dry sample, by means of a calibration curve obtained with Trolox 20–200 µmol/L, in the same assay conditions. The concentration of the sample at a clearance rate of 50% (IC50, μmol/Trolox eq.) was calculated based on the dose–effect curve.

#### 3.10.3. Ferric Reducing Antioxidant Power (FRAP) Assay

FRAP assay was performed to determine the capacity of hazelnut cuticles to reduce Fe^3+^ to Fe^2+^ according to the method of Amrati et al. [56] with slight modifications. 10 μL sample or standard solution, in this case Trolox, was placed in a 96-well microplate followed by the addition of 190 μL FRAP freshly prepared reagent. Then, the absorbance was spectrophotometrically measured at 594 nm in kinetic mode for 60 min at 37 °C using a microplate reader (Cytation 3, Bioteck, Arcugnano, Italy). The experiments were performed in triplicate. The results were expressed as mg Trolox equivalent (TE) per g of dried extract, based on the plotted calibration curve of the standard Trolox in a concentration range of 0.45–100 µmol/L.

#### 3.10.4. Cupric Ion Reducing Antioxidant Capacity (CUPRAC) Assay

CUPRAC was determined according to the method of Apak et al. [57], with small modifications. Briefly, 5 μL of sample, positive control (ascorbic acid), calibration standards (10 mmol/L Trolox stock solution) and blank sample (ethanol) were added to a 96-well microtiter plate in the technical tripsolution (7.5 mmol/L), ammonium acetate solution with pH = 7.0 and distilled water were added in a 1:1:1:1 (*v*/*v*) ratio and was shaken for 12 sec. Then, it was transferred 20 µL of samples into separate wells of a clear, flat bottom 96 well plate. After, 100 µL of Reaction Mix to all assay wells were added, the plate was incubated for 10 min at room temperature. Finally, absorbance was measured at 570 nm using a microplate reader (Cytation 3). CUPRAC values were calculated based on a Trolox calibration curve (y = 0.977x − 0.073 R^2^ = 0.996) within the concentration range 0.25–5.0 nmol/L and the results were expressed as µmol of TE/g dried extract.

### 3.11. Preparation of Milk-like Beverages with the Addition of NADES Extract

Bensmira and Jiang’s method [58] was modified to make nut milk. Roasted hazelnuts (*Corylus avellana* L.) were soaked in water for 12 h and then water is added to the nuts at a ratio of 1:5 (wt/v) to obtain milk with 10 g/100 g total solid content and mixed for 10 min in a blender (Kenwood Thermoresist Glass Blender AT338, Kenwood Europe, New Lane Havant, UK). The resulting mix was filtered by using a cloth bag (standard cotton cloth). The milky liquid obtained was used to produce reference milk (RM). For the production of fortified beverages, NADES extracts were incorporated into the reference milk at concentrations of 5%, 10%, and 20% (*w*/*w*) (NADES-M5, NADES-M10, and NADES-M20, respectively). Considering that hazelnut cuticles account for approximately 2.5% of the total hazelnut weight, the reference milk contained polyphenols deriving from 0.25 g of cuticles per 100 g of milk. The incorporation of NADES extracts (prepared with 0.1 g of freeze-dried roasted hazelnut cuticles per mL of extracting solution) added polyphenols corresponding to 0.5 g, 1 g, and 2 g of cuticles for the 5%, 10%, and 20% formulations, respectively. Milks were subjected to mild pasteurization at 65 °C for 30 min to ensure safety while preserving the bioactive polyphenols. After pasteurization, milks were cooled to room temperature, bottled, and stored at 4 °C until further analysis.

### 3.12. In Vitro Gastro-Intestinal Bioaccessibility

The bioaccessibility of polyphenols in NADES milk or Reference milk was evaluated using the standardized INFOGEST 2.0 protocol established by Minekus et al. [59].

Digestion started with the oral phase where samples (5 mL) were mixed with simulated salivary fluid (SSF, pH 7.0) at a ratio of 1:4 (*v*/*v*). The mixture was incubated at 37 °C for 2 min under gentle agitation. α-Amylase (75 U/mL final activity) was added to simulate enzymatic digestion, and calcium chloride (0.15 mM) was included to maintain ionic strength.

Subsequently, in the gastric phase, the oral bolus (2 mL) was mixed with simulated gastric fluid (SGF, pH 3.0) at a ratio of 1:1 (*v*/*v*) containing pepsin at a final activity of 2000 U/mL, and the mixture was incubated at 37 °C for 2 h with constant shaking at 100 rpm to simulate gastric churning. The pH was monitored and adjusted to 3 as needed using 1 M HCl.

In the intestinal phase, 3 mL of the gastric phase solution was mixed with simulated intestinal fluid (SIF, pH 7.0) at a ratio of 1:1 (*v*/*v*) containing pancreatin (100 U/mL) and bile salts (10 mM), and the mixture was incubated at 37 °C for 2 h under constant agitation. The pH was adjusted to 7.0 using 1 M NaOH to simulate conditions in the small intestine.

After digestion, the samples were centrifuged at 2570 rcf for 10 min to separate the soluble bioaccessible fraction (supernatant) from the non-bioaccessible residue (pellet). The supernatant was collected, filtered, and stored at −20 °C for subsequent polyphenol analysis as described above.

### 3.13. Preference Test

Sensory profiling was conducted by staff from Research Institute on Terrestrial Ecosystem (IRET) with reference to the guidelines in ISO 8586:2014 [60] stating the selection, training, and monitoring of assessors and expert sensory assessors. The Informed written consent was acquired from each participant (10, 40% male and 60% female, aged 24 to 55 years) following the explanation of the experiments. The test was conducted in a climate-controlled room illuminated with white light at around 21 °C. Milk samples (10 mL) were presented in a transparent glass cup, obscured from view and labeled with a random digit code. Samples were presented in a wholly randomized sequence.

Each panelist tasted and evaluated their overall liking using the 7-point hedonic scale followed by the acceptance and preference using the 5-point just-about-right (JAR) scale for 5 attributes: color, milkiness, creaminess, mouthfeel, and aftertaste. For the 7-point hedonic scale evaluating overall liking, panelists rated each sample as 1 = dislike very much, 2 = dislike moderately, 3 = dislike slightly, 4 = neither like nor dislike, 5 = like slightly, 6 = like moderately, and 7 = like very much. For JAR, the attributes and rating description are shown in Table 5.

Data collection was conducted using paper score sheets.

### 3.14. Statistical Analysis

All statistical computations were performed using GraphPad Prism 6.0 software. Statistical differences were assessed using one-way ANOVA, followed by Tukey’s post hoc test for multiple comparisons. Significance levels are indicated as *p* < 0.01, *p* < 0.005, and *p* < 0.001. Each experiment in this study was repeated three times, both biologically and technically.

## 4. Conclusions

This study highlights the potential of roasted hazelnut cuticles as a rich source of polyphenols for functional food development, addressing the growing consumer demand for natural, health-promoting ingredients. By employing LC-MS for precise characterization, we successfully identified key bioactive compounds and optimized their extraction using a biocompatible NADES composed of choline chloride and lactic acid. This innovative approach not only enhanced the extraction efficiency of polyphenols but also demonstrated the sustainability and eco-friendliness of NADESs as an alternative to conventional solvents. The application of the NADES extract in the development of hazelnut-derived vegetable milk further underscored its versatility. The fortified milk exhibited significantly higher polyphenol content and enhanced bioaccessibility compared to conventional extraction methods, without compromising sensory and organoleptic qualities. These findings position NADES-based extractions as a transformative tool for the valorization of food by-products, enabling the creation of nutritionally superior and consumer-acceptable functional foods. The developed hazelnut milk stands as a promising functional food product, capable of delivering high concentrations of bioactive compounds with potential antioxidant and anti-inflammatory benefits, thus aligning with the global shift towards healthier and more sustainable dietary choices.

## Figures and Tables

**Table 1:** Parameters related to NADES synthesis.

Sample *	Molar Ratio (ChCl:HBD)	Synthesis Temperature (°C)	Physical Characteristics	Viscosity at 25 °C (mPa·s)	Viscosity at 50 °C (mPa·s)	Total Phenolic Content (mg GAE/g)
1	1:1	60	Too viscous, less homogeneous	3.24 ± 0.29 ^a^	1.85 ± 0.12 ^b^	137.49 ± 11.03
2	1:2	60	Homogeneous, transparent, stable, viscous	1.56 ± 0.11 ^a^	0.98 ± 0.08 ^b^	160.88 ± 14.27
3	1:3	60	Insufficiently homogeneous, less viscous	0.98 ± 0.08 ^a^	0.62 ± 0.05 ^b^	124.37 ± 9.18
4	1:1	80	Too viscous, less homogeneous	3.11 ± 0.13 ^a^	1.78 ± 0.09 ^b^	125.11 ± 10.43
5	1:2	80	Homogeneous, viscous, stable	1.36 ± 0.07 ^a^	0.79 ± 0.06 ^b^	149.16 ± 13.11
6	1:3	80	Insufficiently homogeneous, less viscous	0.86 ± 0.06 ^a^	0.61 ± 0.04 ^b^	111.96 ± 8.74
7	1:1	60	Too viscous, less homogeneous	7.35 ± 0.49 ^a^	6.81 ± 0.44 ^b^	47.52 ± 4.16
8	1:2	60	Too viscous, less homogeneous	4.51 ± 0.37 ^a^	3.92 ± 0.27 ^b^	60.57 ± 8.32
9	1:3	60	Insufficiently homogeneous	2.76 ± 0.19 ^a^	1.22 ± 0.07 ^b^	136.29 ± 11.18
10	1:1	80	Too viscous, less homogeneous	6.86 ± 0.32 ^a^	5.98 ± 0.29 ^b^	55.49 ± 7.69
11	1:2	80	Too viscous, less homogeneous	4.09 ± 0.25 ^a^	3.07 ± 0.21 ^b^	62.83 ± 9.15
12	1:3	80	Homogeneous, viscous, stable	2.65 ± 0.11 ^a^	0.99 ± 0.08 ^b^	147.56 ± 13.11

* Codes 1–6 refer to ChCl:LA NADESs; Codes 7–12 refer to ChCl:MA NADESs. ^a,b^ Letters are assigned within each line (Group 25–50 °C) to distinguish differences (samples with the same letter are not significantly different).

**Table 2:** Extraction of polyphenols from roasted hazelnut cuticles using different solvents.

Polyphenol	Water 100% (mg/Kg)	Ethanol 20% in Water (mg/Kg)	Ethanol 80% in Water (mg/Kg)	Ethanol 100% (mg/Kg)	NADES 1 (mg/Kg)	NADES 2 (mg/Kg)
Gallic Acid	5212.77 ± 225.11 ^a^	5885.39 ± 254.17 ^a^	8323.62 ± 359.46 ^b^	7566.93 ± 326.78 ^c^	8407.69 ± 363.09 ^d^	8215.39 ± 311.12 ^b^
Caffeic Acid	80.47 ± 7.44 ^a^	90.81 ± 8.41 ^b^	128.51 ± 11.89 ^c^	116.82 ± 10.81 ^d^	129.80 ± 12.01 ^c^	119.81 ± 11.09 ^d^
p-Coumaric Acid	13.33 ± 1.37 ^a^	15.05 ± 1.54 ^a^	21.29 ± 2.18 ^b^	19.35 ± 1.99 ^c^	21.50 ± 2.21 ^b^	19.83 ± 1.89 ^c^
Ferulic Acid	8.81 ± 2.11 ^a^	9.95 ± 2.37 ^a^	14.08 ± 3.36 ^b^	12.81 ± 3.05 ^c^	14.22 ± 3.39 ^b^	13.14 ± 2.88 ^c^
Quercetin-3-*O*-Rutinoside	11.81 ± 0.72 ^a^	13.34 ± 0.82 ^b^	18.87 ± 1.15 ^c^	17.14 ± 1.05 ^d^	19.05 ± 1.16 ^c^	17.65 ± 1.11 ^d^
Catechin	85.41 ± 6.11 ^a^	96.41 ± 6.88 ^b^	136.34 ± 9.76 ^c^	123.95 ± 8.87 ^d^	137.72 ± 9.86 ^c^	129.29 ± 8.74 ^d^
Epigallocatechin	6.19 ± 1.39 ^a^	6.98 ± 1.57 ^a^	9.87 ± 2.23 ^b^	8.98 ± 2.03 ^c^	9.97 ± 2.26 ^b^	9.04 ± 2.39 ^c^
Kaempferol-3-*O*-Glucoside	2.17 ± 0.19 ^a^	2.45 ± 0.22 ^a^	3.47 ± 0.31 ^b^	3.15 ± 0.28 ^c^	3.50 ± 0.31 ^b^	2.92 ± 0.56 ^c^
Quercetin	13.11 ± 5.23 ^a^	14.81 ± 5.91 ^a^	20.93 ± 8.35 ^b^	19.03 ± 7.59 ^b^	21.14 ± 8.43 ^b^	19.31 ± 7.54 ^b^
Apigenin	48.49 ± 3.72 ^a^	54.75 ± 4.19 ^b^	77.43 ± 5.94 ^c^	70.39 ± 5.39 ^d^	78.21 ± 5.99 ^c^	75.22 ± 3.67 ^c^

^a,b,c,d^ Letters are assigned within each line to distinguish differences (samples with the same letter are not significantly different).

**Table 3:** Evaluation of storage.

Sample	DPPH Value Day 0 (µmol Trolox/g Dry Sample)	DPPH Value Day 30 (µmol Trolox/g Dry Sample)	ABTS Value Day 0 (µmol Trolox/g Dry Sample)	ABTS Value Day 30 (µmol Trolox/g Dry Sample)
Reference milk	6113.12 ± 21.41 ^a^	3870.11 ± 11.24 ^b^	81.73 ± 3.42 ^x^	52.64 ± 2.37 ^y^
NADES 5	6227.31 ± 23.94 ^a^	4108.16 ± 14.13 ^b^	85.22 ± 4.11 ^x^	54.92 ± 2.83 ^y^
NADES 10	7894.56 ± 37.25 ^a^	6435.21 ± 25.22 ^b^	127.26 ± 8.34 ^x^	103.82 ± 4.17 ^y^
NADES 20	8194.56 ± 41.36 ^a^	6720.17 ± 31.26 ^b^	146.96 ± 9.87 ^x^	119.54 ± 6.39 ^y^

^a,b^ Letters are assigned within each line to distinguish differences (samples with the same letter are not significantly different). ^x,y^ Letters are assigned within each line to distinguish differences (samples with the same letter are not significantly different).

**Table 4:** Polyphenol bioaccessibility of produced hazelnut milk.

	TPC Reference Milk (mg GAE/g)	TPC NADES-M20 (mg GAE/g)	Bioaccesibility of Reference Milk	Bioaccesibility of NADES-M20
Plain	97.76 ± 19.18 ^a^	2101.92 ± 23.34 ^b^		
Salivary digestion stage	96.94 ± 16.15 ^a^	2099.16 ± 25.13 ^b^	99.2% ^x^	99.8% ^x^
Gastric digestion stage	85.99±15.18 ^a^	1996.30±19.15 ^b^	87.9% ^x^	95.1% ^y^
Intestinal digestion stage	72.15±2.88 ^a^	1790.68±15.63 ^b^	73.8% ^x^	89.7% ^y^

^a,b^ Letters are assigned within each line to distinguish differences (samples with the same letter are not significantly different). ^x,y^ Letters are assigned within each line to distinguish differences (samples with the same letter are not significantly different).

**Table 5:** Attributes and 5-point just-about-right (JAR) scale used.

Sensory Attributes	Rating Description
Color	1. Too Light 2. Slightly Light 3. Just Right 4. Slightly Dark 5. Too Dark
Milkiness	1. Not Milky Enough 2. Slightly Not Milky 3. Just Right 4. Slightly Milky 5. Too Milky
Creaminess	1. Not Creamy Enough 2. Slightly Not Creamy 3. Just Right 4. Slightly Creamy 5. Too Creamy
Mouthfeel	1. Too Thin 2. Slightly Thin 3. Just Right 4. Slightly Thick 5. Too Thick
Aftertaste	1. Too Weak 2. Slightly Weak 3. Just Right 4. Slightly Strong 5. Too Strong

## Data Availability

The data used to support the findings of this study can be made available by the corresponding author upon request.

## Supplementary Materials

The following supporting information can be downloaded at https://www.mdpi.com/article/10.3390/molecules30030433/s1, Table S1. Extraction efficiency of acid-based and sugar-based NADES; Table S2. Analyzed polyphenols.
