# Supplementary material for: Functional Plant-Based Beverage Fortified with Hazelnut Cuticle Polyphenols: Antioxidant and Phenolic Content Characterization
Source: Molecules. 2025 Jan 21;30(3):433. doi: 10.3390/molecules30030433 (PMC11820487; doi:10.3390/molecules30030433)
Supplement: Supplementary file 1 [file molecules-30-00433-s001.zip › molecules-3410390-supplementary.pdf]

**Table S1.** Extraction efficiency of acid-based and sugar-based NADES

| Sample* | Molar Ratio (ChCl:HBD) | Total phenolic content (mg GAE/g) |
|---|---|---|
| ChCl:LA NADES | 1:2 | 160.88 ± 14.27 |
| ChCl:MA NADES | 1:2 | 62.83 ± 9.15 |
| ChCl:CA NADES | 1:2 | 55.16 ± 8.34 |
| ChCl:GL NADES | 1:2 | 49.11 ± 6.11 |
| ChCl:SU NADES | 1:2 | 41.96 ± 7.74 |

LA: Lactic acid, MA: Malic acid, CA: Citric acid, GL: Glucose, SU: Sucrose

**Table S2**. Analyzed polyphenols

| Analyte | m/z |
|---|---|
| Syringic Acid | 198.9 |
| Gallic Acid | 168.9 |
| Quercetin | 301 |
| P Coumarci Acid | 162.9 |
| Oleochantal | 303.2 |
| Hydroxytyrosol | 153.05 |
| Trans Ferulic Acid | 193 |
| Oleuropein | 539 |
| Hesperetin | 301.3 |
| Trimethoxyflavone | 312 |
| Arbutin | 271.2 |
| Rosmarinic Acid | 359 |
| Ursolic Acid | 455 |
| Apigenin | 269 |
| Amentoflavone | 537.1 |
| Luteoilin | 284.9 |
| Quercetin-3-O-Glucoside | 463.1 |
| Quercetin-3-O- Glucuronic Acid | 477 |
| Kaempferol-3-O-Glucose | 609.1 |
| Quercetin-3-O.Hexose Deoxyhexose | 609.1 |
| Isorhamnetin- 3-O Rutinoside | 623.1 |
| Isorhamnetin-7-O- Pentose | 447.1 |
| Luteoilin 7-O-Glucoside | 447.1 |
| Kaempferol-3-O-Glucuronic Acid | 461.1 |
| Kaempferol-3-O-Pentose | 417.1 |
| Kaempferol-3-O-Hexose Deohyhexose | 593.1 |
| Tyrosol | 153.4 |
| Protocathecoic Acid | 153 |
| Vanillic Acid | 167 |
| Syringic Acid | 197 |
| P-Hydroxybenzoic\Salicilic Acid | 137 |
| Gentisic Acid | 153 |
| Caffeic Acid | 179 |
| Sinapic Acid | 223 |
| Ferulic Acid | 193 |
| Trans-Cinnamic Acid | 147 |
| Chlorogenic Acid | 353 |
| Cathechin\Epicathechin | 289 |

| | |
|---|---|
| Gallocathechin\Epigallocathechin Gallate | 457 |
| Gallocathechin\Epigallocathechin | 305 |
| Cathechin Gallate | 441 |
| Procianidin | 577 |
| Myricetin | 317 |
| Kaempeferol | 285 |
| Rutin | 609 |
| Narigin | 579 |
| Licopene | 536.1 |
| Delphinidin3-Rutinoside-5-Galactoside | 772.8 |
| Delphinidin-3-Glucoside | 465.2 |
| Delphinidin-3-Rutinoside | 611 |
| N-Caffeoylputrescine | 249 |
| 3-Caffeoylquinic Acid | 353 |
| Dihydroxycinnamoyl Amide | 470 |
| N,N'-Dicaffeoylspermidine | 468 |

Instrumental settings were configured for a selected ion monitoring (SIM) experiment in negative ion mode, except for syringic acid, lycopene, delphinidin-3-rutinoside-5-galactoside, delphinidin-3-glucoside, and delphinidin-3-rutinoside, which were analyzed using positive electrospray ionization (ESI) mode. Compounds were identified by comparing their characteristic fragmentation patterns with a proprietary molecular database. Identification was confirmed if the area under the curve (AUC) exceeded that of the control blank. Additionally, time-of-flight measurements were employed to distinguish between structurally similar molecules by determining their precise molecular weights in the third quadrupole
